# Eye region surface temperature dynamics during acute stress relate to baseline glucocorticoids independently of environmental conditions

**DOI:** 10.1016/j.physbeh.2019.112627

**Published:** 2019-10-15

**Authors:** Paul Jerem, Susanne Jenni-Eiermann, Dorothy McKeegan, Dominic J. McCafferty, Ruedi G. Nager

**Affiliations:** a Department of Evolution, Behaviour and Environment, School of Life Sciences, University of Sussex, Falmer, UK; b Swiss Ornithological Institute, Sempach, Switzerland; c Institute of Biodiversity, Animal Health & Comparative Medicine, College of Medical, Veterinary & Life Sciences, University of Glasgow, Glasgow, UK

**Keywords:** Bird, Avian, Stress-induced hyperthermia, Homeostasis, Infrared thermography, ‘Fight-or-flight’ response

## Abstract

Reactions to acute stressors are critical for survival. Yet, the challenges of assessing underlying physiological processes in the field limit our understanding of how variation in the acute stress response relates to fitness in free-living animals. Glucocorticoid secretion during acute stress can be measured from blood plasma concentrations, but each blood sample can only provide information for one point in time. Also, the number of samples that can be extracted from an individual in the field is usually limited to avoid compromising welfare. This restricts capacity for repeated assessment, and therefore temporal resolution of findings within- and between-acute stress responses - both of which are important for determining links between acute stress and fitness. Acute stress induces additional body surface temperature changes that can be measured non-invasively, and at high frequencies using thermal imaging, offering opportunities to overcome these limitations. But, this method's usefulness in the field depends on the extent that environmental conditions affect the body surface temperature response, which remains poorly understood. We assessed the relative importance of individual physiology (baseline glucocorticoid concentrations) and environmental conditions (air temperature and relative humidity) in determining the eye region surface temperature (*T*_*eye*_) response to acute stress, in wild blue tits (*Cyanistes caeruleus*) during trapping, handling and blood sampling. When controlling for between-individual baseline variation, *T*_*eye*_ initially dropped rapidly below, and then recovered above baseline, before declining more slowly until the end of the test, 160 s after trap closure. One measure of the amplitude of this response – the size of the initial drop in *T*_*eye*_ – was dependent on environmental conditions, but not baseline corticosterone. Whereas, two properties defining response dynamics – the timing of the initial drop, and the slope of the subsequent recovery – were related to baseline corticosterone concentrations, independently of environmental conditions. This suggests inferring the acute stress response using thermal imaging of *T*_*eye*_ will be practical under fluctuating environmental conditions in the field.

## Highlights

• We used thermal imaging (TI) to infer acute physiological stress in wild blue tits.
• Body surface temperature fluctuated during trapping, handling and blood sampling.
• A rapid initial drop was followed by recovery above baseline, then a slower decline.
• Baseline corticosterone, not environmental conditions predicted two response dynamics.
• Using TI to infer acute stress should be practical in varying environmental conditions.

## 1 Introduction

Stress challenges an organism's homeostasis, and can be either acute (short term), or chronic (long term) [1,2]. Acute stress is common in the natural environment, and reactions to acute stressors, such as predator attack or agonistic social interactions are vital emergency responses, critical for survival [3]. Yet, the challenges in assessing underlying physiological processes in the field limit our understanding of how variation in the acute stress response relates to fitness in free-living animals.

Two functionally linked physiological systems are triggered during acute stress in vertebrates – the sympathetic-adrenal-medullary (SAM) system, and the hypothalamic-pituitary-adrenal (HPA) axis [4,5]. Both release effector hormones (catecholamines and glucocorticoids, respectively), mediating physiological and behavioural changes made to deal with the stressor [6]. The rapidity of the SAM response, and the ephemeral nature of catecholamines [7], make assaying them prohibitively difficult. Glucocorticoid molecules secreted during HPA activity are more stable, and so more readily evaluated [8]. Accordingly, variation in glucocorticoid secretion during acute stress can be measured from blood plasma concentrations. Nonetheless, each blood sample can only provide information for one point in time, and the number of blood samples that can be extracted from an individual in the field is usually limited to avoid compromising welfare. This restricts capacity for repeated assessment, and therefore temporal resolution of findings within- and between-acute stress responses - both of which are important for determining links between acute stress and fitness. For example, aspects of response shape (e.g. negative feedback strength) are emerging as having greater value than single time point measurements (e.g. maximum titres) in predicting fitness [9], while establishing within-individual repeatability between acute stress responses is essential for determining response phenotypes [10].

Acute stress leads to a host of additional physiological adjustments, including changes in respiration and heart rate, that may also be measured as indicators of stress [7]. Telemetry/wireless respiration measurement systems have been successfully employed with captive animals [11,12], and could potentially be used in the wild. But, these methods are currently limited to large species due to technological constraints. And, while progress has been made towards assessing heart rate responses in wild-caught animals via telemetry, the techniques are invasive, involving surgical placement of sub-cutaneous electrodes [[13], [14], [15], [16]]. Both the effects of surgery and the burden of attached instruments can impact specific aspects of physiological responses [17,18], potentially affecting results.

SAM system activation also results in increased core body temperature, known as stress-induced hyperthermia [19,20]. In addition to a centrally regulated elevation of the ‘thermoregulatory set-point’ [21], cutaneous vasoconstriction and shunting by arteriovenous anastomoses [22] diverts blood to core organs with the greatest metabolic need [23]. Simultaneously, reduced blood transport from the warmer core to the cooler periphery decreases body surface temperature [24], proportionally with HPA axis activity (glucocorticoid secretion) [25]. Importantly, such body surface temperature changes can be evaluated without physical contact, and at high measurement frequencies using thermal imaging [[25], [26], [27], [28]]. As a result, thermal imaging offers advantages over other methods in removing welfare restrictions on repeat measurements (so providing increased temporal resolution within- and between-acute stress responses), and affording the possibility of entirely non-invasive sampling.

However, most studies using thermal imaging to investigate acute stress effects on body surface temperature have been conducted in controlled environments, under constant conditions [25,27,[29], [30], [31], [32]]. This is noteworthy as, depending on the level of insulation [33], air temperature can have considerable influence on body surface temperature in endotherms [[34], [35], [36], [37], [38]], and such influence could potentially confound changes in body surface temperature during acute stress. So, little is known regarding how variation in the body surface temperature response to acute stress is partitioned between effects of individual physiology and environmental conditions.

The relative influence of individual physiology and environmental conditions is important in determining the usefulness of thermal imaging for inferring the acute stress response in the wild, especially in locations where environmental conditions vary widely. If the response is primarily driven by physiological processes, and relatively unaffected by environmental conditions, then it should be detectable in a wide range of circumstances. The body surface temperature response to acute stress may relate to individual physiology as a result of allostatic state [2]. As well as being elevated during acute stress, allostatic load fluctuates with the predictable demands of daily life processes, breeding stage, and seasonal environmental changes [39]. Baseline glucocorticoid concentrations are especially important in mediating responses to increased allostatic load [39], and are positively associated with the amplitude of the HPA axis response to acute stress in several wild bird species [[40], [41], [42], [43]]. Glucocorticoid levels also exert influence over the sympathetic nervous system, mediating sensitivity of adrenoreceptors to catecholamines [44]. Consequently, features of the SAM system response to acute stress, such as body surface temperature dynamics, are predicted to relate to baseline glucocorticoid concentrations.

Conversely, If the body surface temperature response to acute stress is constrained by environmental conditions, this could restrict its use as a biomarker in wild populations. At air temperatures outside an individual's thermoneutral zone, peripheral vasomotor control may be used to manage heat loss to the environment, especially in body surface regions used as ‘thermal windows’ [45]. If peripheral blood vessels are already narrowed (or opened) to their full extent, then limited scope for further constriction (or dilation) could restrict the amplitude of stress-related changes in body surface temperature – so-called floor and ceiling effects [46]. Atmospheric humidity may also affect heat transfer to the environment, either through differences between humidity at the body surface and surrounding air driving heat loss by vapour transfer [47], or direct effects on the thermal conductivity of air [48]. Additionally, during procedures which require handling, such as the blood sampling necessary to measure corticosterone concentrations, hand temperature may affect body surface temperature in a similar way to air temperature. Heat transfer between the hand and bird (or vice versa), and the insulating effect of being enclosed within the hand could both lead to peripheral vasomotor responses being made by the bird to regulate body temperature.

Our aim in this study was to assess the relative importance of individual baseline physiology and environmental conditions in determining body surface temperature dynamics during acute stress. To do this, we related body surface temperature, measured from thermal imaging video of free-living blue tits (*Cyanistes caeruleus*) during trapping, handling and blood sampling, to baseline corticosterone concentrations, air temperature, relative humidity and hand temperature. We predicted variation in the body surface temperature response to acute stress would be associated with the individual's baseline corticosterone concentrations, but this relationship could be modified, and potentially masked by effects of air temperature, humidity and hand temperature.

## 2 Methods

### 2.1 Fieldwork

Data collection took place between 10th–13th March 2014 in the Atlantic oak forest surrounding the Scottish Centre for Ecology and the Natural Environment (56.13°N, 4.13°W). Blue tits (*Cyanistes caeruleus*) were caught in walk-in box traps [26] installed at four sites across the study area (0.8 ± 0.2 km apart), and continuously baited with granulated peanuts for ≥1 month prior to trapping. This period was intended to allow birds to habituate to the experimental set-up, and minimise sampling bias towards bold individuals [49,50]. As physiological responses to acute stress are likely to undergo seasonal changes [51] we selected a relatively short sampling period to avoid such variation, while still allowing some fluctuation of environmental conditions (Table 1). Daytime sampling (between 08:18 and 16:52, 12:50 ± 32 min, mean ± SEM, civil twilight start/end; March 10th = 06:10/18:48, March 13th = 06:02/18:54) was chosen to minimise the effect of circadian changes in body temperature [52] and baseline corticosterone levels [53], which are greatest during the transitions between activity and inactivity (and vice versa). All fieldwork was approved by the UK Home Office, and carried out in accordance with the Animals (Scientific Procedures) Act 1986.

**Table 1:** Units, summary statistics and sample sizes of environmental variables and eye region surface temperature (*T*_*eye*_) response properties assessed during trapping, handling and blood sampling (see Fig. 2 and Methods for definitions).

	Environmental variables			*T*_*eye*_ Response properties					
	Temperature		Relative humidity	Amplitude		Dynamics			
	Air	Hand		1. *A*_*drop*_	2. *A*_*recov*_	3. *S*_*drop*_	4. *m*_*recov*_	5. *S*_*recov*_	6. *m*_*decline*_
**Unit**	°C	°C	%	°C	°C	s	–	s	–
**Min**	0.7	23.4	51.2	−3.34	−0.3	1.33	1.57	18.7	−1.1 × 10^−3^
**Max**	11.2	33.1	100.0	−0.19	4.2	19.6	10.31	67.7	−1.0 × 10^−4^
**Mean** **±** **SE**	7.7 ± 0.6	27.9 ± 0.6	87.9 ± 3.0	−1.63 ± 0.15	1.93 ± 0.22	10.33 ± 0.83	−3.98 ± 0.38	41.36 ± 2.72	−5.4 × 10^−4^ ± 4.6 × 10^−5^
**n**	30	30	30	30	30	30	29	30	30

A total of 30 blue tits were trapped. This sample size was chosen to ensure detection of an effect size of 0.65 (Cohen's *d*), calculated from pilot work with captive zebra finches (*Taeniopygia guttata*) (R. Nager, unpublished data), with a power of 80% at *p* = 0.05. Trapping, handling and blood sampling were filmed throughout, using a thermal imaging camera (FLIR A65, f = 25 mm, spatial resolution 0.68 mrad, thermal sensitivity<0.05 °C @ +30 °C, frame rate = 7.5 Hz, FLIR Systems, Wilsonville, Oregon). The camera was positioned 50 cm away from the trap, such that birds were always within the field of view and depth of field, both within the trap, and during handling (see [26] for further details on the trapping and filming set-up).

When a single blue tit entered the trap, it was allowed to feed undisturbed for 4.2 ± 0.16 s to acquire a representative measure of baseline body surface temperature for that individual [26]. The trap was then closed, using a fishing line operated by the experimenter from a concealed position. The trapped bird was subsequently caught by hand (17.4 ± 0.8 s after trap closure), removed from the trap, and held in front of the thermal imaging camera for 223.4 ± 4.2 s. This duration was judged to allow data collection covering the probable period of interest suggested by earlier work [26], and for as long as possible subsequently, while avoiding detrimental effects of prolonged handling (e.g. capture myopathy [54]). During this time, a blood sample (approximately 30 μl) was extracted from the jugular vein by venipuncture (within 112.3 ± 3.2 s of trap closure, range = 78.1–165.1 s), and immediately placed on ice. Plasma was then separated from the blood samples by centrifugation (10 min at 2000 rpm), and used to measure baseline corticosterone concentrations. After completion of filming, the bird's mass (to the nearest 0.1 g) and maximal wing chord (to the nearest 0.5 mm) were measured. Finally, sex was established from plumage characteristics [55], and colour ring combinations were fitted to unringed individuals, prior to release. Each individual was sampled once only. Repeated trapping/sampling was prevented by establishing the colour ring combination of ringed birds prior to shutting the trap, or during handling.

As body temperature and heat loss from the body could be affected by air temperature [56], humidity [57], and hand temperature during handling, these variables were measured during all tests. Air temperature was recorded using a Tinytag Talk 2 Temperature Logger (Gemini Data Loggers UK, Chichester, West Sussex) mounted onto the trap. Relative humidity was recorded every 30 min (Minimet Automatic Weather Station, Skye Instruments, Wales) at the centre of the study site. Hand temperature was recorded from the thermal videos, using the techniques described below.

### 2.2 Thermal video processing

From the thermal videos, bird body surface temperature was measured from the eye region, specifically the exposed skin around the eye (an area comprised of approximately 230 pixels in images recorded using our setup). In the blue tit, the periorbital skin is the only area of the body surface where heat transfer to the environment is not modulated by insulating feathers or leg scales [58,59]. The legs also play a key thermoregulatory role in birds [60], which may obscure effects of acute stress on body surface temperature. For example, if vasoconstriction occurs in the legs to manage heat transfer to the environment, capacity for further constriction during the acute stress response (and resulting reductions in leg surface temperature) could be limited. The legs were therefore not considered as a suitable region of interest for this study. Throughout handling, birds were held with their head parallel to the camera's sensor (i.e. in profile) to ensure eye region surface temperature (*T*_*eye*_) could be measured at all times.

*T*_*eye*_ was extracted from the thermal video using ResearchIR v3.4 (FLIR Systems, Wilsonville, Oregon), and the techniques described in Jerem et al. [26]. For each frame, we extracted the maximum temperature, which almost always occurred within the periorbital ring. In rare cases where the maximum temperature occurred in other locations (such as where a bird's movement caused the plumage to open over an area of muscle), these frames were removed from the analysis. Maximum temperature was used, since thermal imaging is more likely to underestimate than overestimate temperature when targeting small warm passerines on larger cool backgrounds, as a result of motion blur [26]. Overestimation was avoided by shielding the trap interior and the held bird from the sun, effectively preventing energy input into the scene recorded by the camera's sensor. Also, measurement error relating to random drift in sensitivity of individual microbolometers (pixels) on the camera's sensor was minimised by using the camera's ‘non-uniformity correction’ function at ≤3 min intervals while waiting for birds to enter the trap. Remaining microbolometer bias was compensated for by the movement of the bird, our relatively high measurement frequency (7.5 Hz) and the use of maximum temperature (which usually comprised a single pixel). Combined, these factors meant measurements were always spread temporally across multiple microbolometers, effectively averaging between them over the time series. Hence, the maximum temperature measured from the eye region was always likely to be the most accurate measurement recorded. We defined baseline *T*_*eye*_ as the highest value of *T*_*eye*_ recorded in the period an individual spent undisturbed within the trap, prior to trap closure [26].

To further reduce statistical noise relating to underestimation, raw *T*_*eye*_ data were filtered using a ‘peak search’ algorithm to extract local maxima [26]. Additionally, time was standardised between videos by setting trap closure as *t* = 0, and temperature was standardised by subtracting baseline *T*_*eye*_ from test *T*_*eye*_, to control for individual variation in baseline *T*_*eye*_. The combination of filtering of the raw data, and periods where the bird's eye was not visible to the camera resulted in incomplete time series (mean inter-*T*_*eye*_ measurement gap = 3.2 ± 0.3 s). Therefore, to further standardise the data for comparison between individuals, we interpolated each time series to give one temperature value per second per individual using *na.approx* (R package: zoo 1.7–11, [61]).

Hand temperature was extracted as the maximum temperature measured from the palm during the first five seconds after the hand entered the field of view (measurement time 1.95 ± 0.24 s after hand entry). This period was when the palm was most visible, and occurred before substantial hand:bird heat exchange could take place. The palm was chosen as the measurement site, as it is usually the warmest part of the hand in cold conditions [62], and has the largest area of potential contact with the bird during handling. Consequently, the palm was expected to be the most important area for hand:bird heat transfer. Maximum temperature was again used to avoid underestimation resulting from motion blur of the warm hand moving against the cool background of the trap interior.

Both the *T*_*eye*_ data, and the hand temperature measurements were directly calibrated against an object of known temperature (a thermistor probe) and similar emissivity to skin, placed within the field of view of the thermal imaging camera. This method improves accuracy by avoiding the cumulative measurement error inherent when determining the various parameters required by thermal imaging cameras to calculate surface temperature (air temperature, humidity, reflected apparent temperature, distance to camera and emissivity). Instead, these parameters are held constant between the object of known temperature and the target. In small imaging scenes such as ours, the first three are naturally virtually identical, while fixing the thermistor probe onto the front of the trap meant the distance between it and the camera was acceptably consistent with the distance between the target (i.e. the bird or hand within the trap) and the camera. Comparable emissivity with animal integument (>0.94, [91]) was ensured by coating the thermistor probe in black insulation tape (Tesa UK, Milton Keynes, Buckinghamshire, emissivity = 0.97), and emissivity was set at 0.95 across the whole frame in ResearchIR. As such, the relationship between the actual temperature and that estimated by the camera is identical for both the probe and the target. Accordingly, the difference between the temperature logged by the thermistor probe (once per second) and the mean probe temperature estimated by the thermal imaging camera was used to correct *T*_*eye*_ and hand temperature [26].

### 2.3 Glucocorticoid assay

From the blood samples, we assessed total corticosterone concentrations using a commercial ELISA kit (Enzo Life Sciences, Switzerland), validated for the European blackbird (*Turdus merula*), and the great tit (*Parus major*) [63], a close relative of the blue tit. The assay was carried out according to the manufacturer's instructions. One reading was below the detection limit (1 ng/ml), so was set to 0.999 ng/ml. Intra-assay variation was low (e.g. compared to 11.8% reported in Ouyang et al. [63]), ranging from 0.57 to 0.64%, depending on internal control concentration.

### 2.4 Statistical analysis

All data visualisation and analyses were carried out using R v3.1.2 [92]. The mean of all raw and filtered/standardised individual *T*_*eye*_ changes over time during trapping, handling and blood sampling was plotted using *ggplot* (R package: ggplot2 1.0.0, [64]). Basic nonparametric bootstrapped 95% confidence intervals were added to each mean curve using *mean.cl.boot* (R Package: Hmisc 3.14–6, [65]).

To provide detailed characterisation of associations between the baseline-standardised *T*_*eye*_ response to trapping, handling and blood sampling, and environmental conditions/baseline corticosterone concentrations, six distinct properties of the mean response were identified for separate analysis (Fig. 2a). Two properties described the response amplitude - the amplitude of the initial drop in *T*_*eye*_ (*A*_*drop*_, 1. in Fig. 2a) was defined as the lowest value of *T*_*eye*_ recorded within 30 s of trap closure, while the amplitude of the post *A*_*drop*_ recovery (*A*_*recov*_, 2. in Fig. 2a) was defined as the highest *T*_*eye*_ value recorded after *A*_*drop*_. The four remaining properties characterised the response dynamics. The time elapsed (in seconds) between trap closure and *A*_*drop*_ was designated as *s*_*drop*_ (3. in Fig. 2a). As the post-*A*_*drop*_ recovery in *T*_*eye*_ was sigmoidal, its slope *m*_*recov*_ (4. in Fig. 2a) was calculated as *scal* in the non-linear regression equation:

$$y=\mathrm{asym}/\left(1+\exp\left(\mathrm{xmid}-x\right)/\mathrm{scal}\right)$$

where, *x* is time, *y* is *T*_*eye*_, *asym* is the asymptote and *xmid* is the inflection point. *m*_*recov*_ in one individual was more than two standard deviations from the mean, and so was considered an outlier and removed from the analysis; removal of this datapoint had no qualitative effect on the statistical outcome. The number of seconds elapsed between trap closure and *A*_*recov*_ was specified as *s*_*recov*_ (5. in Fig. 2a). Finally, the decline in *T*_*eye*_ following *A*_*recov*_ (*m*_*decline*_, 6. in Fig. 2a) was considered to be exponential, and so was calculated as *k* in:

$$y\left(t\right)=a*e^{\mathrm{kt}}$$

where *a* was *T*_*eye*_ at *A*_*recov*_, and *y(t)* was *T*_*eye*_ at 160 s after trap closure (the latest point at which data was available from all tests).

Relationships between each individual curve property (response variable) and air temperature, humidity, and the individual's baseline corticosterone concentrations (explanatory variables) were analysed using separate multivariate general linear models (GLM). Hand temperature was also included as an additional explanatory variable in models where the response variable was a curve property measured after capture of the bird within the hand.

Baseline corticosterone concentrations and body temperature exhibit circadian rhythms [56,66], and can differ between sexes [3,67], and with body condition [35,68]. Also, thermal conductance to the environment is partly dependent on surface area to volume ratio, which changes with body size [69], and responses to acute stress can be affected by previous experience of handling [70]. Additionally, the time taken to capture the bird in the hand could affect *T*_*eye*_ dynamics during trapping, handling and blood sampling. For example, longer capture times may be associated with greater energy expenditure attempting escape, and so increased heat generation and transfer to the environment. Furthermore, as the possible extent of vasoconstriction/dilation is finite, capacity for heat transfer could be restricted by body temperature immediately before trapping [46]. Therefore, time of day, sex, body condition (calculated as the residuals of an ordinary least squares regression of mass against wing-chord cubed [71]), wing length (a proxy for body size), whether birds had been previously caught (unringed birds were assumed to have been caught for the first time), capture time, and baseline *T*_*eye*_ were all considered as potential confounding explanatory variables. However, time of day and baseline *T*_*eye*_ correlated with air temperature, so simultaneously examining the effects of these variables in our analyses would result in collinearity. As our main interest was to account for environmental and physiological influences, and we restricted measurement to a relatively short period of the day over which diurnal physiological changes are minimal, we included air temperature, but not time of day or baseline *T*_*eye*_ in our analyses.

Potential confounding explanatory variables were only included in full multivariate models if they were associated with the focal curve property in preliminary univariate tests at *p* ≤ 0.1. Based on these preliminary analyses, capture time was included in the *s*_*drop*_ model (*F*_1,29_ = 3.96, *p* = 0.0005), while body condition and previous capture experience were included in the *m*_*decline*_ model (body condition: *F*_1,28_ = 1.98, *p* = 0.06; ringed(yes): *F*_1,28_ = 2.00, p = 0.06). No other potential confounding variables were included in the full models (all *p* > 0.14).

Relationships between corticosterone concentration (response variable) and blood sampling latency (explanatory variable), and hand temperature (response variable) and air temperature (explanatory variable) were assessed using univariate linear models. In the blood sampling latency model, one data point was judged to be an overly influential outlier, having Cook's Distance = 1.0. Therefore robust regression with an MM-type estimator (*lmrob,* R package: ‘robustbase’ v0.93–1.1, [72]) was employed for this analysis.

Significance of specific explanatory variables (critical two-tailed *p* ≤ 0.05) was determined using backwards-stepwise (from most to least complex), pairwise model selection (R function: *drop1*). Reported t and *p* values are from the last model that included a given explanatory variable during the selection process. Effect size *r* of parameters was calculated using equations specified by Nakagawa & Cuthill [73]. All model assumptions were met, and variance inflation factors <3 calculated post-hoc for explanatory variables (*vif*, R package: ‘car’ v2.0–22, [74]) suggested no issue with collinearity in the final models. Throughout the results we report means±standard error.

## 3 Results

**Fig. 1:**
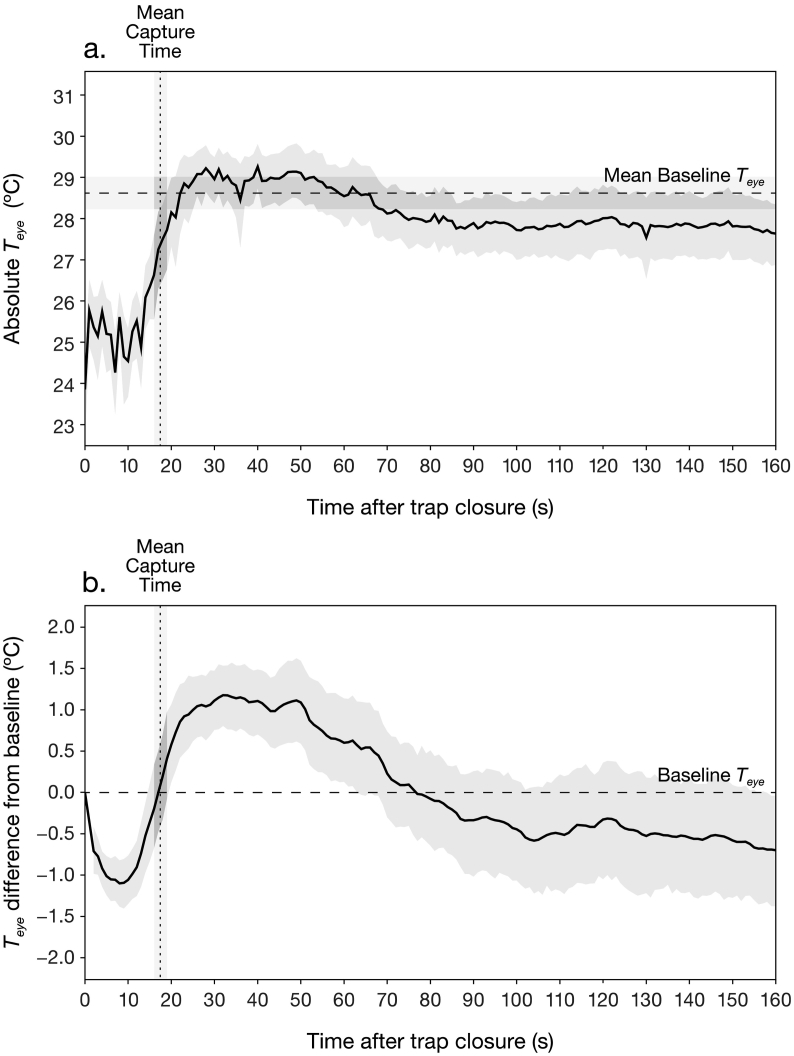
Mean eye region surface temperature (*T*_*eye*_) response to trapping, handling and blood sampling (*n* = 30), as (a) raw, absolute data, and (b) filtered using a ‘peak search’ algorithm (see Methods), and standardised as deviation from baseline. Each individual curve contributing to the mean was interpolated to provide one value per second, per individual (see Methods). Shaded areas indicate 95% confidence intervals. Vertical dotted line marks the mean time point by which the bird was captured in the experimenter's hand.

Baseline *T*_*eye*_ varied between 26.5 and 31.1 (28.6 ± 0.2) °C, while baseline corticosterone concentrations ranged from 0.999–18.61 (8.26 ± 0.66) ng/ml. Blood sampling latency was unrelated to baseline corticosterone concentration (*t*_*29*_ = 1.54, *p* = 0.14). During trapping, handling and blood sampling, absolute eye region surface temperature (*T*_*eye*_) dropped between measurement of baseline and trap closure, returning to baseline levels shortly after capture in the hand (Fig. 1a). In contrast, when individual variation in baseline *T*_*eye*_ was controlled for, a more complex pattern of *T*_*eye*_ change emerged. The baseline-standardised response featured an initial drop in *T*_*eye*_ starting on trap closure, after which *T*_*eye*_ increased, reaching a maximum above the baseline value (Fig. 1b). This was then followed by a slower decline in *T*_*eye*_ over the remainder of the test.

**Fig. 2:**
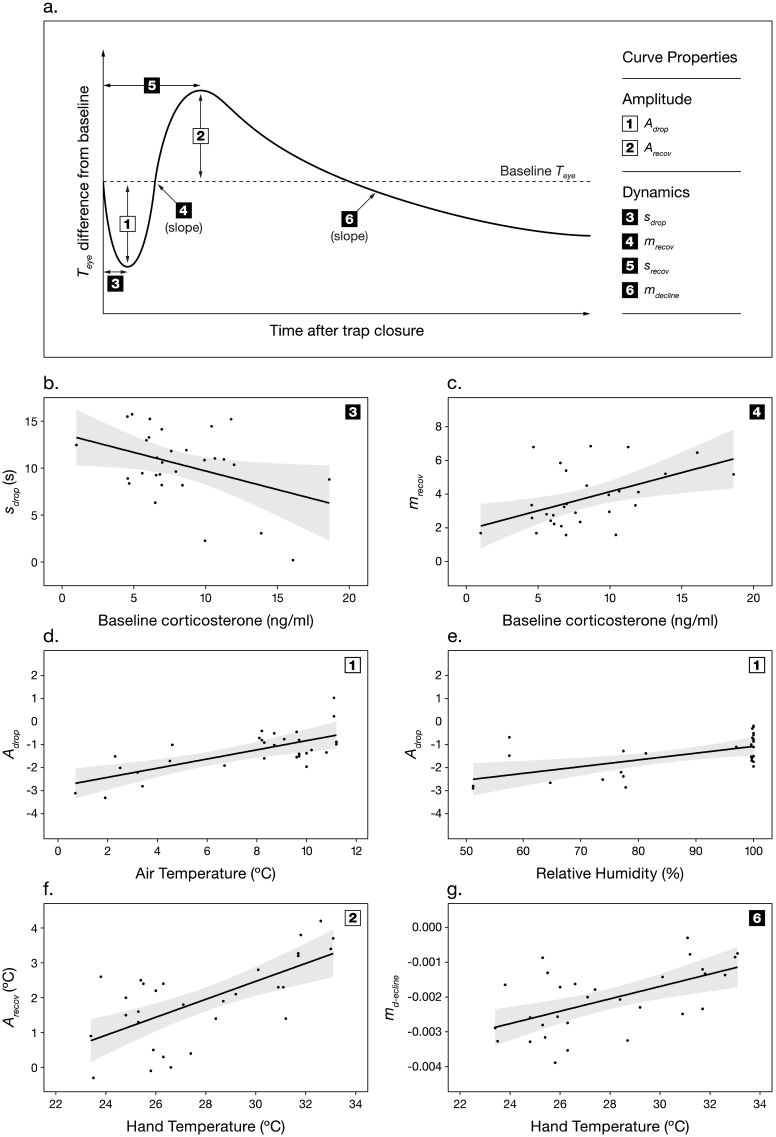
(a) Schematic baseline-standardised eye region surface temperature (*T*_*eye*_) response to trapping, handling and blood sampling, identifying separate curve properties analysed (see Methods for definitions). Also, GLM model predictions of relationships between response curve properties and baseline corticosterone (b) & (c), air temperature/relative humidity (d) & (e), and hand temperature (f) & (g). Predictions are conditional on other parameters in the models (see Table 2) being set to their median value. Shaded areas indicate 95% confidence intervals.

Of the distinct properties of the baseline-standardised *T*_*eye*_ response (Fig. 2a, Table 1), *s*_*drop*_ (the interval between trap closure and initial drop minimum) was shorter, and *m*_*recov*_ (the slope of the subsequent recovery) was less steep with increasing baseline corticosterone concentrations (Fig. 2b & c, Table 2). *S*_*drop*_ was also shorter in birds that were caught in the hand more quickly, but neither *s*_*drop*_ or *m*_*recov*_ were related to environmental conditions (Table 2). In contrast, the amplitude of the initial drop in *T*_*eye*_ at its lowest point (*A*_*drop*_) was only related to environmental conditions; *A*_*drop*_ was greater at lower air temperatures and relative humidities, and was independent of baseline corticosterone concentrations (Fig. 2d & e, Table 2).

**Table 2:** GLM model summaries relating properties of the baseline-standardised *T*_*eye*_ response to trapping, handling and blood sampling (see Fig. 2 and Methods for definitions) with baseline corticosterone, environmental variables, and hand temperature. Other covariates (capture time, body condition and previous capture experience - whether an individual was already ringed) were included in models only if found to relate to the curve property in preliminary univariate tests (critical *p* ≤ 0.1). Coefficient estimates are presented ±95% confidence intervals, and *r* is the parameter effect size. Model R^2^ is the proportion of variation in the response variable explained by the final model. Significant relationships are highlighted in bold.

	Amplitude								Dynamics															
	1.*A*_*drop*_				2.*A*_*recov*_				3.*S*_*drop*_				4.*m*_*recov*_				5.*S*_*recov*_				6.*m*_*decline*_			
	Estimate	t	p	*r*	Estimate	t	p	*r*	Estimate	t	p	*r*	Estimate	t	p	*r*	Estimate	t	p	*r*	Estimate	t	p	*r*
**Intercept**	−5.74 ± 2.21	5.07	<0.0001	–	−5.26 ± 2.31	3.42	0.002	–	3.83 ± 2.21	1.40	0.17	–	1.88 ± 1.39	2.65	0.01	–	−3.36 ± 48.02	0.14	0.89	–	−7.0 × 10^−3^ ±2.6 × 10^−3^	5.29	<0.0001	–
**Baseline Corticosterone**	0.03 ± 0.07	0.93	0.36	0.18	−0.04 ± 0.11	0.73	0.47	0.14	−**0.40** **±** **0.35**	**2.23**	**0.03**	**0.39**	**0.23** **±** **0.15**	**2.90**	**0.008**	**0.49**	−0.29 ± 1.70	0.34	0.74	0.14	1.3 × 10^−4^ ±8.6 × 10^−5^	0.30	0.76	0.06
**Air Temperature**	**0.20** **±** **0.10**	**3.95**	**0.0005**	**0.61**	0.09 ± 0.13	1.28	0.21	0.24	0.06 ± 0.58	0.19	0.85	0.04	−0.22 ± 0.23	1.84	0.08	0.35	−0.09 ± 2.58	0.07	0.95	0.19	−7.4 × 10^−5^ ±8.4 × 10^−5^	1.72	0.10	0.32
**Humidity**	**0.03** **±** **0.02**	**3.09**	**0.005**	**0.51**	0.01 ± 0.02	1.35	0.11	0.25	0.04 ± 0.35	1.05	0.08	0.20	0.02 ± 0.03	0.97	0.34	0.17	0.07 ± 0.32	0.46	0.65	0.12	5.2 × 10^−6^ ±2.5 × 10^−5^	0.41	0.69	0.09
**Hand Temperature**	–	–	–	–	**0.26** **±** **0.11**	**4.69**	**0.00006**	**0.66**	–	–	–	–	–	–	–	–	1.60 ± 1.71	1.84	0.08	0.02	**1.8** **×** **10**^**−4**^ **±9.3** **×** **10**^−**5**^	**3.76**	**0.0008**	**0.58**
**Capture Time**	–	–	–	–	–	–	–	–	**0.75** **±** **0.34**	**4.29**	**0.0002**	**0.64**	–	–	–	–	–	–	–	–	–	–	–	–
**Body Condition**	–	–	–	–	–	–	–	–	–	–	–	–	–	–	–	–	–	–	–	–	5.4 × 10^−4^ ±5.4 × 10^−4^	1.96	0.06	0.35
**Ringed (Yes)**	–	–	–	–	–	–	–	–	–	–	–	–	–	–	–	–	–	–	–	–	2.8 × 10^−4^ ±6.0 × 10^−4^	0.89	0.34	0.09
**Model R**^**2**^	0.37				0.42				0.48				0.21				0.08				0.31			

Once the bird was enclosed in the experimenter's hand (17.4 ± 0.7 s after trap closure), both *A*_*recov*_ and *m*_*decline*_ (the amplitude of the post *A*_*drop*_ recovery, and the rate of cooling during the post-recovery decline, respectively) increased with hand temperature (Fig. 2f & g, Table 2). Hand temperature was not associated with air temperature (*F*_1,28_ = 1.08, *p* = 0.31).

## 4 Discussion

Eye region surface temperature (*T*_*eye*_) changed with a characteristic pattern during the acute stress of trapping, handling and blood sampling. Absolute values of *T*_*eye*_ dropped between measurement of baseline (in the 4.2 ± 0.16 s preceding trap closure) and closure of the trap, returning to baseline after capture in the experimenter's hand. However, controlling for individual variation in baseline *T*_*eye*_ revealed a more complex pattern of *T*_*eye*_ change; after trap closure, *T*_*eye*_ rapidly dropped below, and then increased above baseline, before decreasing more slowly until the end of the test (160 s after the trap closed). One measure of the baseline-standardised response amplitude – the size of the initial drop in *T*_*eye*_ (*A*_*drop*_) – was dependent on environmental conditions (range: air temperature = 0.7–11.2 °C, relative humidity = 51.2–100%), but not baseline corticosterone. Whereas, two properties defining dynamics of the baseline-standardised response – the timing of the initial drop (*s*_*drop*_), and slope of the subsequent recovery (*m*_*recov*_) – were related to baseline corticosterone concentrations independently of environmental conditions. This suggests inferring the acute stress response using thermal imaging of *T*_*eye*_ will be practical under fluctuating environmental conditions in the field.

Baseline corticosterone concentrations we observed were higher than most values measured from blue tits [[75], [76], [77], [78]]. This may relate to the severity of conditions during winter 2014, which was the wettest recorded in Scotland at that time [79]. Nonetheless, sampling latency was unrelated to our measure of corticosterone concentrations. So, our values were not influenced by the acute stress of trapping, handling and blood sampling, and should represent true baselines. Published baseline body surface temperature data from free-living birds is scarce, but baseline *T*_*eye*_ values recorded during this study were lower than those reported from the same population during the breeding season [35]. This difference most likely relates to lower winter air temperatures, although it remains possible seasonal variation in metabolic processes and energy reserves also played a role [35].

The additional detail revealed by analysing *T*_*eye*_ as difference from baseline (in contrast to using absolute values) highlights the utility of this method in exposing the complex pattern of *T*_*eye*_ change during acute stress in statistically noisy thermal imaging data. The overall shape of the baseline-standardised *T*_*eye*_ response to trapping, handling and blood sampling differs from that observed in the same population during trapping without handling/blood sampling [26]. Both responses shared a rapid initial drop of similar duration and amplitude, and ended just below baseline after 160 s. But, the recovery period in *T*_*eye*_ following the initial drop was shorter in non-handled birds, and did not exceed baseline *T*_*eye*_. The 95% confidence intervals for the two mean responses overlap between zero and approximately 15 s after trap closure, and again at the end of the test [80]. The similarity in initial *T*_*eye*_ changes demonstrates this aspect of the eye region surface temperature response to acute stress is a reproducible phenomenon. While peripheral vasoconstriction causes the initial drop in *T*_*eye*_, the subsequent increase above baseline in trapped, handled and blood sampled birds is likely to be related to a range of non-mutually exclusive factors. Firstly, the *T*_*eye*_ increase between *A*_*drop*_ and *A*_*recov*_ may result from processes aimed at regulation of a new, higher thermoregulatory set-point for core body temperature induced by acute stress [21]. Secondly, handling appears to have warmed or insulated the bird, as the amplitude of the post- *A*_*drop*_ rise in *T*_*eye*_ (*A*_*recov*_) increased with hand temperature. If heat was being transferred from the hand to the bird, it would also be expected that warmer hand temperatures would be associated with lower rates of cooling (*m*_*Decline*_) after *A*_*recov*_, but the opposite was observed. This may indicate active thermoregulatory effort by the bird to avoid overheating. Regardless, these effects highlight the necessity of accounting for hand temperature in future work involving handling. The differences in overall response shapes may also relate to seasonal changes in the acute stress response [51], as this study was carried out shortly before the breeding season (March), while the ‘trapping without handling’ data was collected mid-winter (December–January). Lastly, response shape could reflect the intensity of acute stress. Variation in body surface temperature responses between different stressors has previously been linked with stressor intensity [25,81]. In chickens, the response in comb temperature to a less intense stressor showed an initial drop, and subsequently remained below baseline whereas the response to a more intense stressor exhibited an increase above baseline after the initial drop [25]. Therefore trapping (i.e. confinement) could be a less intense stressor than trapping, handling and blood sampling in blue tits, as observed in some other bird species ([82], although see [83]).

The extent of body surface temperature changes we observed are within the magnitude of those seen in other bird species subjected to handling [25,29,84]. Immediate drops in body surface temperature (similar to those reported here but longer in duration) were also observed from a variety of alternative measurement sites in both chicken (*Gallus gallus domesticus*) studies [25,29]. Larger species are slower to change internal body temperatures due to the low thermal diffusivity of tissues [85], while smaller species have higher surface area to volume ratios that allow for greater heat transfer to the environment per unit volume [86]. Both factors may contribute to the more rapid initial drop in blue tits compared to larger birds. However, direct comparison of response shape with these studies is difficult, as methods, regions of interest, and test durations differed, and measurement frequencies were substantially lower than those achieved here. Additionally, the species investigated (budgerigar *Melopsittacus undulatus* and chicken) are domesticated, which can modulate responses to acute stress [87].

Two dynamic properties of the *T*_*eye*_ response measured before the birds were caught in the hand (*s*_*drop*_ and *m*_*recov*_) were related to baseline corticosterone concentrations, but not to air temperature or humidity. Both associations exhibit effect sizes that would be considered ‘medium’ (i.e. not small) [88]. These relationships suggest a link between pre-stressor allostatic state and peripheral temperature changes during acute stress, which is not affected by variation in environmental conditions – at least within the range we measured. Quicker initial *T*_*eye*_ responses (i.e. shorter *s*_*drop*_) in individuals with higher baseline corticosterone suggest these birds had a higher stress reactivity. The initial drop in *T*_*eye*_ (*A*_*drop*_) occurs due to sympathetically mediated diversion of blood away from the periphery to internal organs [23,24]. As such, the slower recovery (higher *m*_*recov*_) in birds with higher baseline corticosterone may be further evidence of a stronger initial reaction, with blood supply having been diverted to internal organs for longer. In contrast to the dynamic properties, neither measure of *T*_*eye*_ response amplitude was related to baseline corticosterone. Given the capacity of glucocorticoids to influence the sympathetic nervous system [44], this lack of relationships is somewhat surprising. However, the intensity of interactions between the HPA-axis and SAM system is highly variable, and depends on numerous factors including stressor intensity [4,5]. Therefore, it is possible that remaining unexplained variation in *T*_*eye*_ response amplitude is related to stress-induced, rather than baseline corticosterone concentrations. Alternatively, response amplitude may be more sensitive to environmental variation than dynamics, masking expected relationships with baseline corticosterone.

While unrelated to baseline corticosterone, *A*_*drop*_ was dependent on environmental conditions, increasing with lower air temperature and humidity. The main driver of this association is most likely air temperature, as humidity should only have a marginal effect on the thermal conductivity of air at the temperatures we recorded [48]. Notably, if the relationship between air temperature and *A*_*drop*_ continues to be linear at temperatures above those we recorded, it might be expected that no drop in *T*_*eye*_ would occur above around 15 °C. This would limit the usefulness of *T*_*eye*_ in inferring the acute stress response in the field, even in temperate zones. However, such an outcome seems improbable, given two previous studies report substantial (approximately 0.5–2 °C) initial drops in chicken surface temperature during handling at air temperatures of 18 °C and 23 °C, respectively [25,29]. Nevertheless, where Lewden et al. [89] observed a drop in cloacal temperature (measured once at 2.3 ± 1.7 min, and then again at 12.6 ± 4.2 min after stressor onset) in response to trapping, handling and blood sampling at low temperatures (−6.6 ± 0.04 °C), they found no difference in warmer conditions (18.7 ± 0.09 °C). Whether this actually represents an absence of body temperature response is unclear though, as only two measurements of temperature were taken at time points that would have missed the dynamics we captured by measuring multiple times per second from the moment of stressor onset. That larger drops in *T*_*eye*_ occurred in colder conditions runs counter to the prediction that ability to restrict blood flow to the body surface in response to stress will be reduced at low temperatures. Vasoconstriction may occur in cold conditions to minimise heat loss [90]. Because there is a finite capacity for vasoconstriction, this should lead to smaller stress-induced *T*_*eye*_ reductions at lower temperatures. As we observed larger *A*_*drop*_ at lower temperatures, an alternative explanation may be that the increased gradient between body surface and air temperatures in cold conditions permits greater heat loss. But, steeper gradients might also be expected to result in more rapid heat loss, generating a negative relationship between air temperature and *s*_*drop*_, which we did not find.

The positive association between *s*_*drop*_ and capture time suggests blood flow remained diverted from the body surface for longer with increased escape behaviour duration. As escape behaviour increases metabolic demands in muscles and core organs, it seems likely this lengthened redirection of blood flow was implemented to meet these needs. It is also possible, though, that blood flow to the surface is reduced during periods of immediate physical risk to minimise the potential for blood loss through wounding [23]. Interestingly, the relationship between *s*_*drop*_ and capture time also implies vasoconstriction ceased on, or slightly before capture in the hand, possibly indicating the end of the initial ‘emergency’ SAM response once capture rendered escape effort futile. But, *s*_*drop*_ in this study was not significantly different to that measured during trapping without handling (*p* = 0.09) [80], suggesting similarities between *s*_*drop*_ and capture time may only be coincidental.

Finally, *m*_*Decline*_ was not related to body condition, or previous experience of handling. Jerem et al. [35] reported a positive relationship between body condition and *T*_*eye*_ in undisturbed blue tits, but the mechanisms linking these traits might not affect acute stress-induced surface temperature dynamics. Also, while Lynn et al. [70] observed reduced HPA responses in Eastern bluebirds (*Sialia sialis*) that had experienced the same blood sampling protocol weeks before, birds in this study with experience of handling had only been ringed previously, sometimes years before. Therefore, future stress responses may only be affected by previous experiences above a certain threshold of stressor intensity, and/or within a limited timeframe.

Our analyses show that within the range of air temperatures we measured, two dynamic properties of the *T*_*eye*_ response to trapping, handling and blood sampling were related to baseline corticosterone concentrations, independently of air temperature and humidity. Consequently, thermal imaging of body surface temperature could provide a useful new method of investigating acute stress in natural environments with fluctuating conditions. Thermal imaging offers advantages over current methods in providing increased temporal resolution, permitting improved response phenotyping by removing welfare limits on repeat measurements, and opening up the possibility of entirely non-invasive sampling. Situations where subject movement during acute stress is limited, such as when nesting birds or cryptic species are exposed to predation risk or human disturbance, would be particularly amenable to a wholly non-invasive approach. While the range of air temperatures in which we conducted our tests was relatively narrow (0.7–11.2 °C), other studies carried out at lower and higher temperatures also report body temperature changes in response to acute stress [25,29,89]. Nevertheless, investigations into whether *T*_*eye*_ dynamics during acute stress remain independent of environmental conditions beyond those measured here would be a desirable next step. In addition, studies comparing *T*_*eye*_, corticosterone concentrations, and other sympathetically mediated traits (e.g. heart/breath rate) throughout the acute stress response, or selectively activating/deactivating underlying systems (e.g. blocking catecholamine reception) could usefully shed light on controlling mechanisms. Also, determining whether multiple pixel maxima are more representative than the single pixel maximum temperatures used in this study may prove worthwhile in further optimizing temperature extraction from thermal video recordings.

## Author contributions

PJ, RGN, DMcK, and DMcC contributed to experimental design. PJ gathered the data. SJE conducted the hormonal assays. PJ analysed the data and wrote the manuscript. All authors contributed critically to the drafts and gave final approval for publication.

## Funding

This work was funded by a Doctoral Training Programme studentship awarded to PJ by the Biotechnology and Biological Sciences Research Council (grant number BB/J013854/1).

## Data accessibility

Data available at https://doi.org/10.5525/gla.researchdata.849.

## Declaration of Competing Interest

We have no competing interests to declare.
